# Prognostic implication of heterogeneity and trajectory progression induced by enzalutamide in prostate cancer

**DOI:** 10.3389/fendo.2023.1148898

**Published:** 2023-03-16

**Authors:** Yuanfa Feng, Yulin Deng, Zhenfeng Tang, Shanghua Cai, Jinchuang Li, Ren Liu, Jiaming Wan, Huichan He, Guohua Zeng, Jianheng Ye, Zhaodong Han, Weide Zhong

**Affiliations:** ^1^ Urology Key Laboratory of Guangdong Province, The First Affiliated Hospital of Guangzhou Medical University, Guangzhou Medical University, Guangzhou, Guangdong, China; ^2^ Department of Urology, Guangdong Key Laboratory of Clinical Molecular Medicine and Diagnostics, Guangzhou First People’s Hospital, School of Medicine, South China University of Technology, Guangzhou, Guangdong, China; ^3^ Guangzhou Medical University, Guangzhou Laboratory, Guangzhou, Guangdong, China; ^4^ Guangdong Provincial Institute of Nephrology, Nanfang Hospital, Southern Medical University, Guangzhou, Guangdong, China; ^5^ Macau Institute for Applied Research in Medicine and Health, Macau University of Science and Technology, Avenida Wai Long, Taipa, Macau, China

**Keywords:** prostate cancer, enzalutamide, relapse-free survival, signature, endocrine therapy

## Abstract

**Background:**

Enzalutamide, as a second-generation endocrine therapy drug for prostate cancer (PCa), is prominent representative among the synthetic androgen receptor antagonists. Currently, there is lack of enzalutamide-induced signature (ENZ-sig) for predicting progression and relapse-free survival (RFS) in PCa.

**Methods:**

Enzalutamide-induced candidate markers were derived from single-cell RNA sequencing analysis integrating three enzalutamide-stimulated models (0-, 48-, and 168-h enzalutamide stimulation). ENZ-sig was constructed on the basis of candidate genes that were associated with RFS in The Cancer Genome Atlas leveraging least absolute shrinkage and selection operator method. The ENZ-sig was further validated in GSE70768, GSE94767, E-MTAB-6128, DFKZ, GSE21034, and GSE70769 datasets. Biological enrichment analysis was used to discover the underlying mechanism between high ENZ-sig and low ENZ-sig in single-cell RNA sequencing and bulk RNA sequencing.

**Results:**

We identified a heterogenous subgroup that induced by enzalutamide stimulation and found 53 enzalutamide-induced candidate markers that are related to trajectory progression and enzalutamide-stimulated. The candidate genes were further narrowed down into 10 genes that are related to RFS in PCa. A 10-gene prognostic model (ENZ-sig)—IFRD1, COL5A2, TUBA1A, CFAP69, TMEM388, ACPP, MANEA, FOSB, SH3BGRL, and ST7—was constructed for the prediction of RFS in PCa. The effective and robust predictability of ENZ-sig was verified in six independent datasets. Biological enrichment analysis revealed that differentially expressed genes in high ENZ-sig were more activated in cell cycle–related pathway. High–ENZ-sig patients were more sensitive to cell cycle–targeted drugs (MK-1775, AZD7762, and MK-8776) than low–ENZ-sig patients in PCa.

**Conclusions:**

Our results provided evidence and insight on the potential utility of ENZ-sig in PCa prognosis and combination therapy strategy of enzalutamide and cell cycle–targeted compounds in treating PCa.

## 1 Introduction

Prostate cancer (PCa), which represents 27% of all new cancer cases every year, is among the most common cancer in men (1). It has been reported that approximately 268,490 cancer cases were diagnosed as PCa and 34,500 deaths were estimated as PCa in the United States in 2022 (1). Despite that diverse treatments including radical prostatectomy, radiotherapy, chemotherapy, and endocrine therapy were leveraging to counter PCa, prognosis and treatment are still poor, especially for the patients who possess high-grade disease (2). As an androgen-dependent disease, endocrine therapy that targets androgens or androgen receptors (AR) had been the first-line therapy for those patients with PCa who did not benefit from surgery or radiation (3). However, whether the endocrine therapy targets the hypothalamic-pituitary negative feedback pathway, inhibits androgen synthesis, or blocks androgen receptors, long-term hormone deficiency would cause a series of side effects, such as hyperlipidemia, osteoporosis, insulin resistance, anemia, and sexual dysfunction (4). More importantly, in response to androgen deprivation, most PCa progresses to castrate-resistant PCa (CRPC), which is inevitable in endocrine treatment for PCa (2). Thus, it is highlighted that how to effectively address the limitations of endocrine therapy is essential to increase the therapeutic efficiency and improve prognosis of patients with PCa.

Enzalutamide, a prominent representative among synthetic AR antagonists, is a second-generation AR antagonist displaying effective antineoplastic by binding directly to the AR. However, sustained medicine with enzalutamide would inevitably progress to enzalutamide resistance and treatment failure (3). Therefore, it is especially significant to improve the therapeutic efficiency of enzalutamide treatment for shortening duration of enzalutamide treatment and prolonging survival. However, the underlying mechanism induced by enzalutamide is still unclear. It is reported that enzalutamide plays the part of agonist in transcriptional activity, inducing the expression of cancer-related genes in PCa cells (5). Moreover, it is noticed that PCa was reported as a heterogeneity tumor that result in distinct cellular phenotypes, and such inter-tumoral heterogeneity would generate different treatment response phenotypes (6, 7). Thus, exploring the enzalutamide-mediated transcription in single-cell resolution may contribute to understand the molecular mechanisms of enzalutamide, even the mechanisms of enzalutamide resistance, which could improve prognosis, and provide potentially strategy of combination treatment for PCa.

In this work, we explored the cell heterogeneity and transcriptional alteration that induced by enzalutamide and identified a heterogeneous cluster that resists enzalutamide-treatment by using single-cell RNA sequencing (scRNA-seq) analysis. Following the time trajectory analysis in 0-, 48-, and 168-h enzalutamide-stimulated models, time-dependent enzalutamide-induced gene sets were further discovered. Then, a prognostic model, named ENZ-sig, was built on the basis of 10 prognostic enzalutamide-induced markers for the prediction of relapse-free survival (RFS) in PCa. We examined the predicting ability and clinical significance of ENZ-sig in PCa from The Cancer Genome Atlas (TCGA-PRAD) and further validated in six independent cohorts (i.e., GSE70768, GSE94767, E-MTAB-6128, DFKZ, GSE21034, and GSE70769). Finally, pathway enrichment analysis revealed that patients in the high-risk group were more activated in cell cycle pathway, giving insight on the combination treatment strategy related to enzalutamide and cell cycle–targeted compounds for patients with PCa.

## 2 Materials and methods

### 2.1 Data sources and processes

The raw count data of scRNA-seq underlying enzalutamide-stimulated LNCap after 0, 48, and 168 h, respectively, were obtained from Taavitsainen et al. (8). The RNA sequencing data and clinical features from TCGA were downloaded and processed by GDCRNAtools (9). The clinical information and mRNA expression data from GSE70768, GSE94767, DFKZ, GSE21034, and GSE70769 datasets were acquired from Gene Expression Omnibus (GEO; https://www.ncbi.nlm.nih.gov/geo/ ), and data from E-MTAB-6128 were derived from ArrayExpress (https://www.ebi.ac.uk/arrayexpress/ ). The detailed information of above datasets is summarized in **Table S1**.

### 2.2 Single-cell RNA sequencing data processing and analysis

By using Seurat package (10), the raw count data were initialized and created as a Seurat object with the criteria of min.cells = 3 and min.features = 200. Then, quality control was performed to ensure that mitochondrial counts were less than 20% and that unique feature counts were less than 5,000 and more than 200 in each cell. Furthermore, standard pre-processing workflow, including data normalization and scaling, and the detection of highly variable features were conducted in the three enzalutamide-induced models, separately. The joint analysis of these three models was further performed by FindIntegrationAnchors() and IntegrateData() with parameters of anchor.features = 2,000 and dims = 1:50. Selecting the top 50 principal components and a resolution of 0.2, the integrated object was clustered into different cell subgroup. Non-linear dimensional reduction was using to visualize and explore the integrated clusters. Differentially up-expressed markers in each cell subgroup were defined using FindAllMarkers() function with log [fold change (FC)] > 0.25 and adjusted p-value < 0.05. Differentially expressed genes (DEGs) in C7 were identified by FindMarkers() function with the cutoff criteria of log|FC|>0.25 and adjusted p-value < 0.05.

### 2.3 Cell trajectory analysis

Monocle R package (v2.24.0) was performed to explore the time trajectory of enzalutamide-induced cell in three stimulated models (DMSO, 48-h stimulation, and 168-h stimulation). The DEGs that derived from three stimulated models were defined as time trajectory features to ordering cells in pseudo-time. The time trajectory markers was obtained from the function of differential GeneTest() in Monocle (11).

### 2.4 Pathway enrichment analysis

To detect the biological changes in cell cluster, pathway score was measured by gene set variation analysis (GSVA) on the basis of the gene sets of “Hallmark” and “KEGG” obtained from MSigDB (12). Differential expression analysis was carried out to explore the DEGs between C7 and other clusters. The statistically significant DEGs with the threefold were identified and further fitted into “enrichR” package. Gene set enrichment analysis (GSEA) was conducted on the basis of the pre-ranking gene set that ordered by the FC from differentially expressed analysis.

### 2.5 Construction and validation of enzalutamide-induced signature

Candidate enzalutamide-induced genes were identified from the gene set that intersected with DEGs in C7 and markers in cell trajectory analysis. To select the key prognostic enzalutamide-induced features, least absolute shrinkage and selection operator (LASSO) method with 10-fold cross-validation was further performed to narrow down the significant enzalutamide-induced genes. Enzalutamide-induced signature (ENZ-sig) was calculated as follows: β(feature1) × expr (feature1) + β(feature2) × expr (feature2) + ··· + β (feature n) × expr (feature n), where β is the LASSO Cox coefficient of each feature in the regression model and expr is the expression value of the corresponding feature. This strategy was further utilized in six independent datasets to validated the efficiency and robustness of ENZ-sig in PCa.

### 2.6 Development and validation of nomogram

A predicting nomogram leverages the vital prognostic characteristics for predicting 3-, 5-, and 7-year RFS of patients with PCa in the TCGA-PRAD dataset. To determine the capability of the nomogram to predict 3-, 5-, and 7-year RFS outcomes, the calibration curves were developed by “rms” R package.

### 2.7 Scoring the activity of ENZ-sig in single cell

To estimate the activity of ENZ-sig for individual cells, R package AUCell was performed on the basis of the expression of 10 ENZ-sig genes (13). The area under the curve (AUC) that generated by AUCell represented the score activity of ENZ-sig in single cell. Cells were divided into two groups on the basis of optimal cutoff value evaluated by AUCell.

### 2.8 Statistical analysis

All statistical analyses in our study were performed in R (version 4.0.5). Kaplan–Meier survival analysis was performed using “survival” R package, and the optimal cutoff value was defined by “survminer”. Wilcoxon signed-rank test, with two-tailed P < 0.05 being considered as statistical significance level. P-value adjustment was applied when multiple comparisons were necessary.

## 3 Results

### 3.1 Landscape of enzalutamide-induced cell heterogeneity and transcriptional alteration

**Figure 1:**
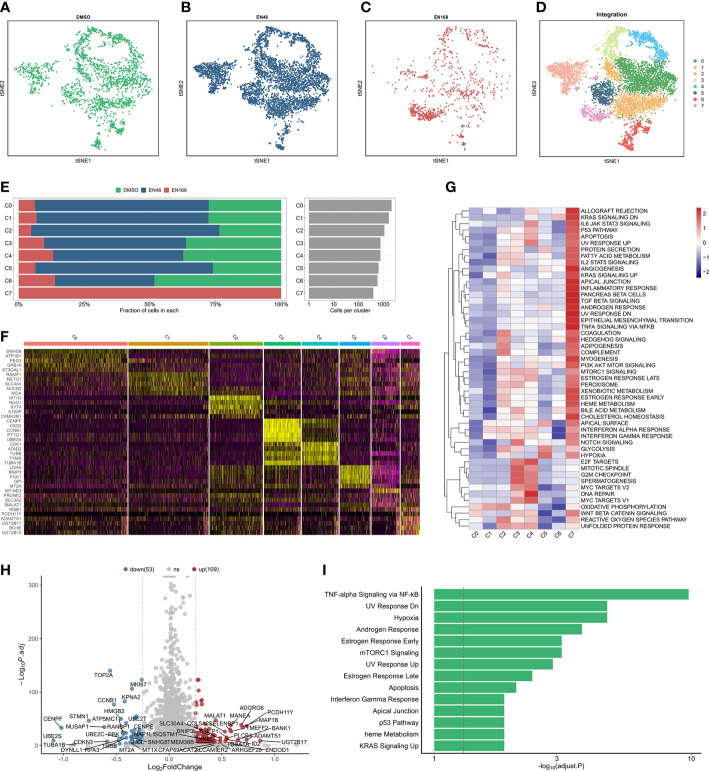
The landscape of integrated profiling induced by enzalutamide. The t-distributed stochastic neighbor embedding (tsne) diagram of cells stimulated: DMSO **(A)**, 48-h enzalutamide **(B)**, and 168-h enzalutamide **(C)**. **(D)** The tsne diagram of integrated analysis induced by enzalutamide in 0, 48, and 168 **(H, E)** The proportion and number of cells in each cluster. **(F)** Heatmap for the top five markers in each cluster. **(G)** Gene set variation analysis in each cluster using Hallmark gene set. **(H)** volcano plot for the differentially expressed genes (DEGs) between cluster 7 and other clusters. Red dots indicate upregulated genes with log_2_(fold change) > 0.25 and adjusted p-value < 0.05. Blue dots represent downregulated genes with log_2_(fold change) < −0.25 and adjusted p-value < 0.05. **(I)** Biological enrichment using the statistically significant genes from DEGs in cluster 7. Gray vertical dotted line indicates the threshold of adjusted p-value less than 0.05.

After quality control and normalization, the dimension reduction on the three types of enzalutamide-derived models is shown in **Figures 1A–C**. The joint analysis of these three models was further performed to explore the characteristics of enzalutamide-induced cell heterogeneity or transcriptional alteration in PCa cell line. Eight clusters were obtained after scRNA-seq integration (**Figure 1D**). Interestingly, by comparing the proportion of originating cells and the number of cells in each cluster, we found that cluster 7 (C7) was mostly enriched in ENZ168 model (**Figure 1E**). Moreover, the top five differentially expressed markers of each cluster were displayed as a heatmap plot (**Figure 1F**). Biological pathway analysis indicates that the enrichment pathway in C7 was more activated in 39 of the 50 Hallmark gene sets compared with other clusters, such as fatty acid metabolism, TGF-β signaling, androgen response, epithelial–mesenchymal transition, and PI3K-AKT-MTOR signaling (**Figure 1G**). The differentially expressed analysis between C7 and other clusters was highlighted that 109 upregulated genes and 53 downregulated genes were significantly differentially expressed in C7 (**Figure 1H**). The upregulated genes were enriched in TNF-α signaling *via* NF-kB, hypoxia, androgen response, and mTORC1 signaling, indicating that C7 displayed strongly tumor-promoting progression under treating with enzalutamide (**Figure 1I**).

### 3.2 Identifying enzalutamide-induced pseudo-time trajectory

**Figure 2:**
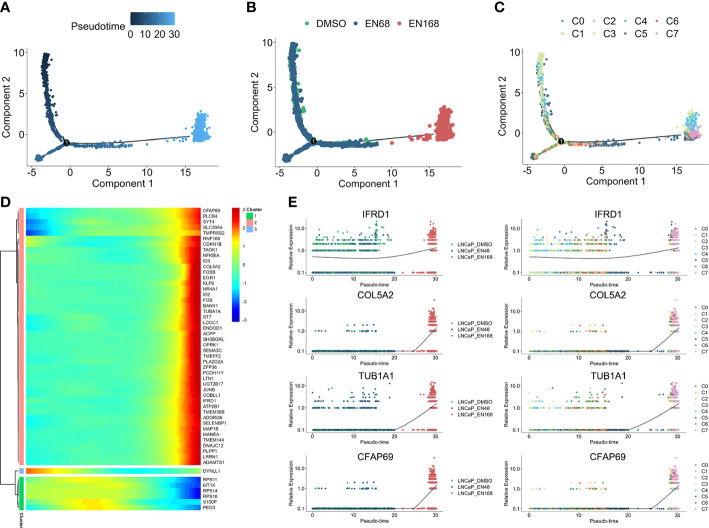
The progression trajectory of enzalutamide-induced and pseudo-time gene expression pattern. Pseudo-trajectory **(A)**, cell type **(B)**, and cell clusters **(C)** of enzalutamide-induced cells. **(D)** Heatmap for expression pattern of genes that induced by enzalutamide-stimulated alone with trajectory-evolved. **(E)** Expression tendency of representative genes in enzalutamide-induced model and cell clusters.

For exploring the enzalutamide-induced gene sets, we further conducted the pseudo-time trajectory analysis to construct a linear trajectory from 0-, 48-, to 168-h enzalutamide-stimulated models (**Figures 2A, B**). As shown in **Figure 2C**, we found that C7 was specifically located at the end of time-trajectory, indicating that C7 was heterogeneous cluster induced by enzalutamide-stimulated alone with trajectory-evolved. Next, 53 dysregulated genes based on the trajectory progression and enzalutamide-stimulated models were detected (**Figure 2D**). Five representative trajectory-related markers (IFRD1, COL5A2, TUB1A1, and CFAP69) show the upregulated expression tendency alone with enzalutamide-stimulated models and transferred cluster (**Figure 2E**).

### 3.3 Construction of enzalutamide-induced signature for predicting RFS in prostate cancer

**Figure 3:**
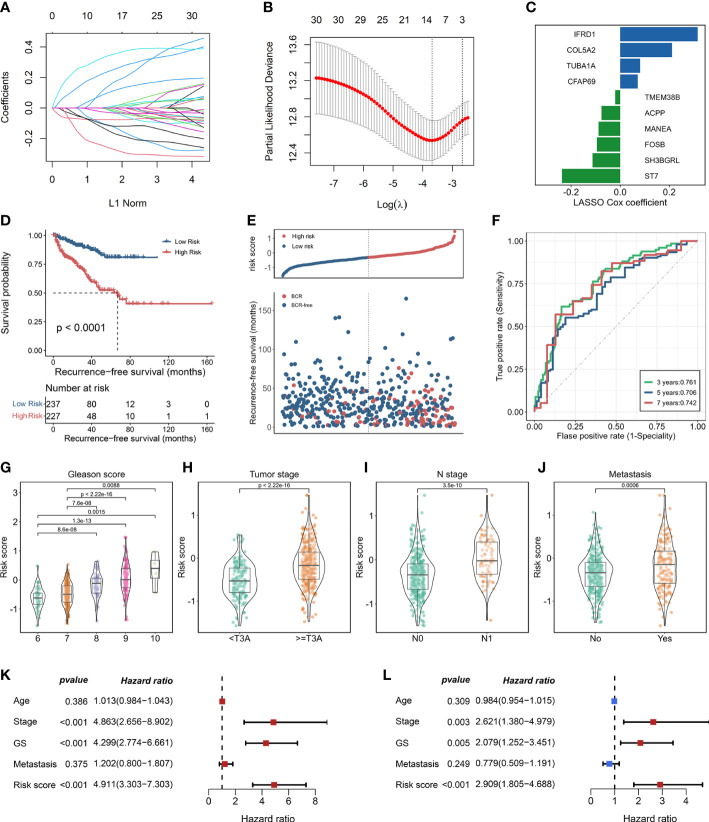
Construction of enzalutamide-induced signature and its clinical relevance. **(A)** Lasso cox regression coefficients of 31 candidate genes. **(B)** Partial likelihood deviance of candidate genes derived from lasso Cox regression analysis with 10-fold cross-validation. Two vertical dotted lines represent minimum mean cross-validation error and one standard error of the minimum, respectively. **(C)** The coefficients of 10 genes selected from lasso Cox regression model. **(D)** Kaplan–Meier curve in terms of RFS for high-risk and low-risk patients divided by enzalutamide-induced signature. **(E)** The risk score and recurrence-free survival status based on TCGA-PRAD cohort. **(F)** The receiver operating characteristics curve for the prediction of 3-, 5-, and 7-year RFS based on the risk score derived from enzalutamide-induced signature. The abundance of risk score calculated by enzalutamide-induced signature in different clinicopathological characteristics, including Gleason score **(G)**, tumor stage **(H)**, N stage **(I)**, and metastasis status **(J)**. Univariate Cox regression analysis **(K)** and multivariate Cox regression analysis **(L)** for enzalutamide-induced signature and clinical variables, including age, stage, Gleason score, and metastasis status.

Thirty-one enzalutamide-induced candidate genes were further generated by intersecting with DEGs in C7 and trajectory-related markers. Lasso algorithm was performed to screen the significantly prognostic genes that induced by enzalutamide in these candidate genes (**Figure 3A**). With the optimal lambda value of 0.025 based on minimal mean square error (**Figure 3B**), 10 genes [i.e., IFRD1, COL5A2, TUBA1A, CFAP69, TMEM38B, ACPP, MANEA, FOSB, SH3BGRL, and suppressor of tumorigenicity (ST7)] were identified as significantly enzalutamide-induced genes that are related to the prognosis of PCa RFS. Meanwhile, IFRD1, COL5A2, TUBA1A, and CFAP69 served as the risk factors (coefficient > 0), and TMEM388, ACPP, MANEA, FOSB, SH3BGRL, and ST7 served as protective factors (coefficient < 0) in PCa (**Figure 3C**). Then, an enzalutamide-related signature was constructed for the prediction of PCa RFS on the basis of 10 genes expression abundances and lasso Cox coefficient. The calculation formula is as follows: 0.316 × expression of IFRD1 + 0.211 × expression of COL5A2 +0.081 × expression of TUBA1A + 0.071 × expression of CFAP69 + (−0.020 × expression of TMEM38B) + (−0.076 × expression of APCC) + (−0.088 × expression of MANEA) + (−0.095 × expression of FOSB) + (−0.113× expression of SH3BGRL) + (−0.237 × expression of ST7). We divided the patients into high- and low-risk groups on the basis of the ENZ-sig and found that patients with higher ENZ-sig presented worse RFS than those with lower ENZ-sig (p < 0.0001, **Figure 3D**). Moreover, the distribution of the risk score and the RFS status are displayed in **Figure 3E**. It is clear that the higher the risk score, the more inclined to relapse. The ROC analysis indicated that the prognostic accuracy values for 3-, 5-, and 7-year RFS were 0.761, 0.706, and 0.742, respectively, for ENZ-sig (**Figure 3F**).

### 3.4 Evaluating the clinical relevance of ENZ-sig in prostate cancer

To explore the clinical relevance of ENZ-sig, we further investigated the relationship between ENZ-sig and clinicopathological features, including Gleason score, tumor stage, N stage, and metastasis. The result revealed that ENZ-sig was significantly positively correlated with Gleason score (**Figure 3G**), tumor stage (**Figure 3H**), N stage (**Figure 3I**), and metastasis (**Figure 3J**), indicating that the levels of ENZ-sig were significantly related to the progression of PCa. The univariate (**Figure 3K**) and multivariate (**Figure 3L**) Cox regression model were further performed to discover the prognostic value of ENZ-sig in PCa. We found that tumor stage (HR = 4.863; 95% CI 2.656 to 8.902; p < 0.001), Gleason score (HR = 4.299; 95% CI, 2.774 to 6.661; p < 0.001), and ENZ-sig (HR = 4.911; 95% CI, 3.303 to 7.303; p < 0.001) were significantly associated with PCa RFS. Moreover, tumor stage (HR = 2.621; 95% CI, 1.380 to 4.979; p = 0.003), Gleason score (HR = 2.079; 95% CI, 1.252 to 3.451; p = 0.005), and ENZ-sig (HR = 2.909; 95% CI, 1.805 to 4.688; p < 0.001) were the independent prognostic factors for predicting RFS in Pca, and its predicting abilities were unrestricted with the existing clinical variables, demonstrating the clinical utility of ENZ-sig for predicting RFS in PCa.

### 3.5 Validation of the enzalutamide-induced signature in independent datasets

**Figure 4:**
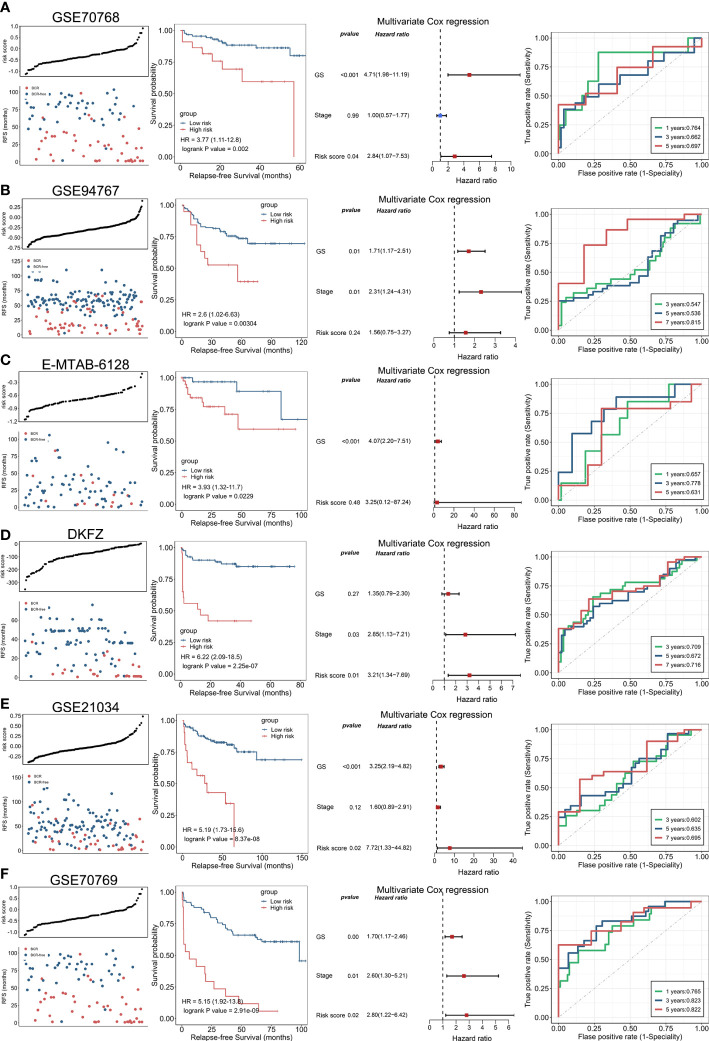
Validation of enzalutamide-induced signature in independent cohort. The plot of risk score and RFS status, Kaplan–Meier curve, multivariate Cox regression analysis, and receiver operating characteristics curve for evaluating the predicting ability of enzalutamide-induced signature in GSE70768 **(A)**, GSE94767 **(B)**, E-MTAB-6128 **(C)**, DFKZ **(D)**, GSE21034 **(E)**, and GSE70769 **(F)** cohorts, respectively.

Following the same calculation formula, we leveraged six independent datasets (GSE70768, GSE94767, E-MTAB-6128, DFKZ, GSE21034, and GSE70769) to verify the robustly predicting performance of ENZ-sig for PCa RFS (**Figures 4A–F**). As expected, PCa RFS was correlated with ENZ-sig, and patients with higher risk scores presented significantly worse RFS compared with those with lower risk scores in the six PCa datasets (GSE70768: HR = 3.77, p = 0.002; GSE94767: HR = 2.6, p = 0.00304; E-MTAB-6128: HR = 3.93, p = 0.0229; DKFZ: HR = 6.22, p = 2.25e-07; GSE21034: HR = 5.19, p = 8.37e-08; and GSE70769: HR = 5.15, p = 2.91e-07). Moreover, the multivariate Cox regression analysis revealed that ENZ-sig was a significantly independent prognostic factor in GSE70768 (HR = 2.84, p = 0.04), DKFZ (HR = 3.21, p = 0.01), GSE21034 (HR = 7.72, p = 0.02), and GSE70769 (HR = 2.80, p = 0.02) cohorts. Importantly, the ROC analysis showed a stable predicting ability of ENZ-sig for PCa RFS in these six cohorts, highlighting that ENZ-sig was a reliable and effective predictor for PCa RFS.

### 3.6 Development of the nomogram and evaluation of its clinical utility

**Figure 5:**
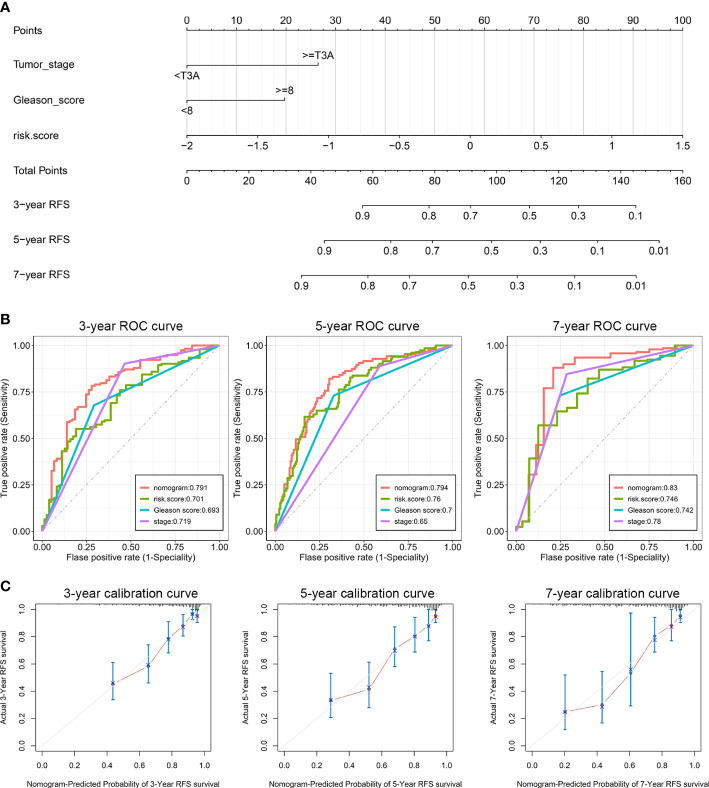
Clinical utilization of enzalutamide-induced signature. Nomograms **(A)**, including the ROC curves **(B)** and the calibration plots **(C)** for the prediction of relapse-free survival for patients with PCa at 3, 5, and 7 years, respectively.

On the basis of our findings that the ENZ-sig calculated by ENZ-sig as well as Gleason score and tumor stage are predictive of PCa RFS, we constructed nomograms for predicting patients’ 3-, 5-, and 7-year RFS, respectively (**Figure 5A**). **Figure 5B** shows that the AUC values of the nomograms were 0.791, 0.794, and 0.83 for 3-, 5-, and 7-year RFS, respectively. Nomogram showed higher AUC values than Gleason score, tumor stage, and ENZ-sig, indicating that the predictability of RFS was improved by integrating these prognostic features. In **Figure 5C**, the calibration curves suggested that the nomogram-predicted probability is relatively close to the actual RFS outcome (diagonal line).

### 3.7 Functional characteristics related to enzalutamide-induced signature

**Figure 6:**
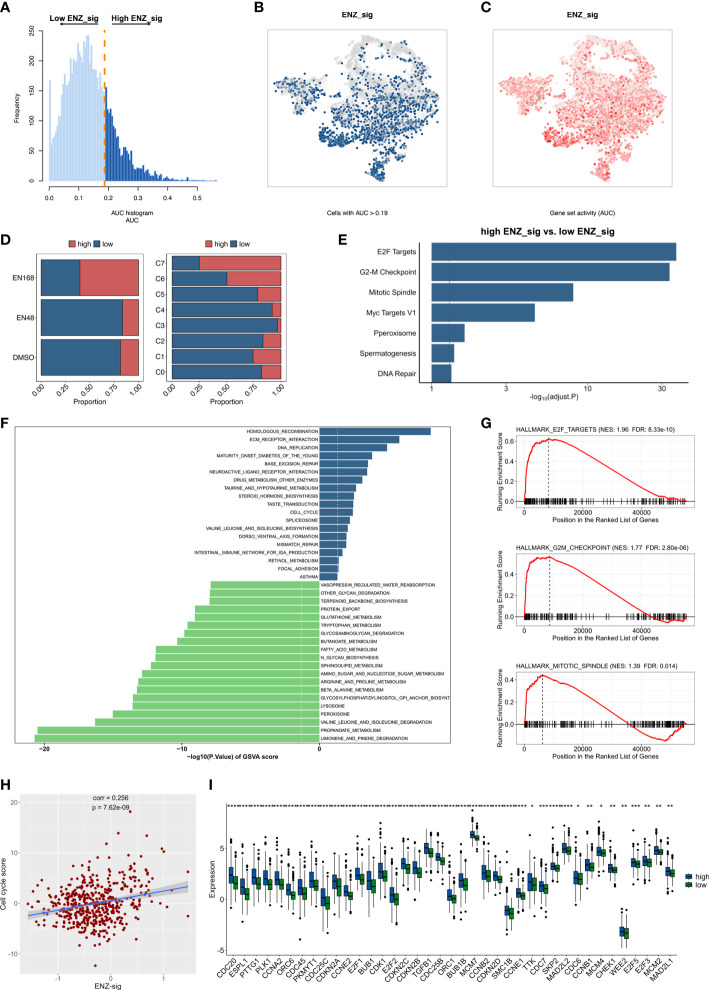
Biological pathway enrichment related to enzalutamide-induced signature. **(A)** The distribution of AUC and cell frequency. **(B)** AUC and cell frequency. **(B)** T-distributed stochastic neighbor embedding (Tsne) diagram for cells with AUC higher than selected threshold. Blue dots indicate cells with higher than 0.19, and gray dots indicates cells with less than 0.19. **(C)** Tsne diagram for ENZ-sig activity that colored by AUC. The deeper color represents a larger AUC. **(D)** The distribution of high ENZ-sig cells and low ENZ-sig cells in enzalutamide-stimulated models and clusters. **(E)** Bar plot for enrichment analysis based on the differentially expressed genes between high ENZ-sig and low ENZ-sig group. Red dotted line represents adjusted p-value less than 0.05. **(F)** Gene set variation analysis between high-risk and low-risk group stratified by enzalutamide-induced signature. Dotted line indicates p-value less than 0.05. **(G)** Gene set enrichment analysis in the high-risk group and the low-risk group. **(H)** Correlation between ENZ-sig and cell cycle score in PCa. **(I)** Box plot shows the statistically significant genes related to cell cycle pathway between the high-risk and low-risk groups. Statistical significance: *p < 0.05; **p < 0.01; ***p < 0.001; ****p < 0.0001.

To discover the biological mechanisms involving the ENZ-sig, we first evaluated the ENZ-sig levels of each cell according to the expression of 10 ENZ-sig genes by AUCell method. It is noticed that AUCell, a scoring system, uses the AUC to measure the score of gene set. The histogram presented the distribution of ENZ-sig levels and cellular frequency (**Figure 6A**). With the optimal threshold estimated by AUCell, cells were divided into high–ENZ-sig and low–ENZ-sig groups. The distribution of the cells with AUC > 0.19 (**Figure 6B**) and ENZ-sig activity (**Figure 6C**) was shown on the t-distributed stochastic neighbor embedding diagram. As expected, the proportion of high–ENZ-sig group was higher in EN168 model and C7 than that in the other models or clusters (**Figure 6D**). Then, biological enrichment analysis was performed on the basis of the DEGs between high ENZ-sig and low ENZ-sig. As shown in **Figure 6E**, cell cycle–related pathways, i.e., E2F targets, G2M checkpoint, mitotic spindle, and Myc targets, were significantly enriched in the high–ENZ-sig group, demonstrating the strong relationship between ENZ-sig and cell cycle. We further conducted the GSVA analysis and compared the differential pathway score between the high-risk and low-risk patients in TCGA-PRAD. The result showed that patients with higher score of ENZ-sig were more activated in the cell cycle pathway than patients with lower score of ENZ-sig (**Figure 6F**). The GSEA was further verified the relation between ENZ-sig and cell cycle pathway. As shown in **Figure 6G**, cell cycle–related pathways, i.e., E2F targets (NES = 1.96, FDR = 8.33e-10), G2M checkpoint (NES = 1.77, FDR = 2.80e-06), and mitotic spindle (NES = 1.39, FDR = 0.014), were significantly enriched in the high-risk group. The correlation analysis revealed the significantly positive relation between ENZ-sig and cell cycle score in PCa [correlation coefficient (corr) = 0.256, p = 7.62e-09; **Figure 6H**], indicating that ENZ-sig mediated the activation of cell cycle pathway that contributed to increasing risk of progression and poor outcome for PCa. Moreover, cell cycle–related genes, e.g., CDC20, PLK1, CDC45, CDK1, CDKN2C, MCM2, E2F5, and E2F3, were significantly upregulated in high–ENZ-sig group (**Figure 6I**).

### 3.8 Ability of model in predicting drug sensitivity

**Figure 7:**
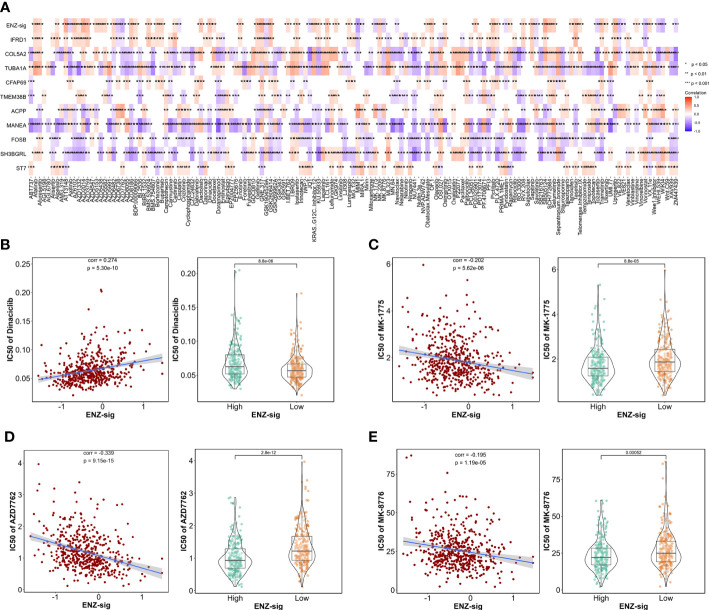
Ability of model in predicting drug sensitivity. **(A)** Heatmap plot indicates the correlation between drugs/compounds, ENZ-sig, and 10 ENZ-signature genes. Scatter diagram and box plot show the relationship between the sensitivity of dinaciclib **(B)**, MK-1775 **(C)**, AZD7762 **(D)**, MK-8776 **(E)**, and ENZ-sig.

Given the significantly prognostic value of ENZ-sig in the prediction of PCa RFS, we further explore the relationship between ENZ-sig and drug sensitivity. First, we measured the half-maximal inhibitory concentration (IC50) of each drug/compound in TCGA-PRAD dataset by “oncoPredict” package. Then, correlation analysis was performed between IC50 and ENZ-sig. The overview result is displayed in **Figure 7A**. We noticed that four cell cycle–targeted drugs, i.e., dinaciclib, MK-1775, AZD7762, and MK-8776, were significantly related to ENZ-sig. In detail, the sensitivity of dinaciclib was positively correlated with ENZ-sig (corr = 0.274, p = 5.30e-10; **Figure 7B**). The IC50 of dinaciclib was higher in high–ENZ-sig group than that in low–ENZ-sig group (p = 8.8e-06; **Figure 7B**). Moreover, in **Figures 7C–E**, MK-1775 (corr = −0.202, p = 5.62e-06), AZD7762 (corr = −0.339, p = 9.15e15), and MK-8776 (corr = −0.195, p = 1.19e-05) presented a significantly negative correlation with ENZ-sig. Furthermore, patients in the high–ENZ-sig group have lower IC50 of MK-1775 (p = 8.8e-05), AZD7762 (p = 2.8e-12), and MK-8776 (p = 0.00052) than those in the low–ENZ-sig group, indicating that patients with higher ENZ-sig were more sensitive with cell cycle–targeted drug/compound in PCa.

## 4 Discussions

The extensive application of scRNA-seq technologies facilitates the comprehension of cell heterogeneity and genomic characteristics that mediated by drug treatment (14). However, enzalutamide-mediated transcriptional activity in scRNA-seq resolution has not been obtained. Leveraging scRNA-seq analysis, we integrated the three enzalutamide-simulated models (i.e., DMSO, ENZ-48, and ENZ-168) and distinguished eight sub-clusters. Notably, unlike other clusters, the proportion of cells in C7 was largely derived from ENZ-168 model. Furthermore, the underlying biological pathway in C7 was more activated in androgen response, epithelial–mesenchymal transition, fatty acid metabolism, and PI3K/AKT/MTOR signaling than those in the other cell clusters, indicating that C7 was a tumor-promoting cluster evoking by enzalutamide. For further exploring the related genes that induced by enzalutamide, time trajectory analysis was performed following the enzalutamide-stimulated timeline (0, 48, and 168 h). We identified several cancer-related genes that presented marked dynamic expression pattern corresponding to enzalutamide-stimulated timeline.

The intersected gene set between differentially expressed markers in C7 and dynamically changed genes in time trajectory analysis was further leveraged to constructed risk model. Thirty-one candidate genes were screened and selected by lasso Cox regression model, and 10 significant enzalutamide-induced genes (IFRD1, COL5A2, TUBA1A, CFAP69, TMEM388, ACPP, MANEA, FOSB, SH3BGRL, and ST7) related to PCa RFS were remained. It is reported that IFRD1 (known as interferon-related developmental regulator 1) encoded a protein related to interferon-γ and was significantly correlated with survival outcome in colon cancer (15). COL5A2 promotes proliferation and invasion in PCa and is related to the prediction of RFS for patients with PCa (16). Moreover, COL5A2 was identified as enzalutamide-resistant genes in CRPC cells *in vitro* (17). Wang et al. showed that TUBA1A, known as tubulin α 1a, was a potential prognostic marker and therapeutic target in gastric cancer (18). CFAP69 has been demonstrated to be a prognostic marker that is related to the survival of breast cancer patients (19). TMEM38B, as regulators of endoplasmic reticulum (ER) calcium storage, was induced by KLF9, contributing to the release of calcium from ER, aggravation of ER stress, and molecular death (20). ACPP (prostate acid phosphate) has shown to be a prognostic factor for predicting the RFS in PCa and to be correlated with CRPC bone metastases (21). MANEA is the sole endo-acting glucoside hydrolase related to N-glycan trimming and disrupting N-linked glycosylation as therapeutic agents for cancer (22). Barrett et al. reported that FOSB is required for migration and invasion in PCa cells (23). Kwon et al. indicated that the declining expression of SH3BGRL was related to the aggressiveness of PCa (24). ST7 was demonstrated to function as tumor suppressor in PCa by remodeling tumor microenvironment (25). Collectively, except for the five genes (COL5A2, ACPP, FOSB, SH3BGRL, and ST7) that function as important biomarkers in PCa, the significant role of another five genes (IFRD1, TUBA1A, CFAP69, TMEM38B, and MANEA) in PCa is still not demonstrated, which needs further investigation. Whereas, all these 10 enzalutamide-induce genes likely play a significant role in PCa and may serve as biomarkers for prognosis of the disease. Interestingly, we noticed that IFRD1 presented the highest lasso Cox coefficient among these 10 genes, suggesting that IFRD1 was making the most vital contribution compared with other nine genes.

Given the vitally prognostic role of these 10 enzalutamide-induced genes, we constructed a predicting model, ENZ-sig, based on transcriptional expression and lasso Cox coefficient of each gene. ENZ-sig presented robust ability to classify patients into high-risk group and low-risk group with significantly different RFSs. Although several risk stratification signatures have been developed for predicting RFS of PCa. Hu et al. reported an overall survival (OS)–related signature for predicting PCa OS based on the expression levels of five autophagy-related genes (ARGs) and a disease-free survival (DFS)–related signature for the prediction of PCa DFS based on the expression of 22 ARGs (26). Mei et al. developed an m7G-related prognostic signature for the prediction of PCa RFS leveraging the data from TCGA and GEO (27). Feng et al. screened and selected 10 genes for establishing circadian clock related signature as a promising tool for the prediction of PCa RFS (28). However, the predicting accuracy and robustness of validation limited their clinical utilization, and, more importantly, enzalutamide-related signature for predicting PCa RFS has not been described yet. In our study, the ENZ-sig was effectively validated in six independent datasets (i.e., GSE70768, GSE94767, E-MTAB-6128, DFKZ, GSE21034, and GSE70769). In these datasets, ENZ-sig has successfully stratified patients into two groups, and the statistical significance in RFS between these two groups was found, strongly indicating the effective and robust prognostic ability of ENZ-sig for predicting PCa RFS.

We further extended the clinical utilization of ENZ-sig by examining the correlation between ENZ-sig and FDA-approved drugs for PCa. The result showed that ENZ-sig was significantly negatively correlated with the IC50 of three cell cycle–targeted drugs (i.e., MK-1775, AZD7762, and MK-8776), suggesting that patients with high ENZ-sig are more sensitive to these cell cycle–targeted drugs than those with low ENZ-sig. It is reported that the combination of enzalutamide and Chk1/2 inhibitor AZD7762 presented additive and synergistic therapeutic effects in xenograft and patient-derived tumor xenograft models *in vivo* (29). Moreover, MU380, a more effective analog of Chk1 inhibitors MK-8776, significantly enhances the sensitivity of human docetaxel-resistant PCa cells to gemcitabine through inducing mitotic catastrophe (30). Furthermore, Bridges et al. illustrated that MK-1775, a novel Wee1 kinase inhibitor, could promote the sensitivity of radiotherapy for p53-defective human tumor cells (30). Given the impressive role of these three cell cycle inhibitors in PCa treatment, ENZ-sig may serve as a clinical indicator not only for the prognosis in RFS but also for supporting the clinical evaluation of cell cycle–targeted drugs in combination therapy for PCa.

Our results provided evidence and insight on the potential utility of ENZ-sig in PCa prognosis and clinical use. However, there are some limitations in the current ENZ-sig model. More enzalutamide-induced genes ought to be discovered to optimize the ENZ-sig model. In addition, the 10 enzalutamide-induced markers identified in the study warrant further experimental investigation to unfold their vital functions in PCa and to explore new therapies targeting these molecules.

## 5 Conclusions

By integrating scRNA-seq and bulk RNA sequencing analysis, we demonstrated a heterogeneous sub-cluster that induced by enzalutamide and identified ENZ-sig for the prediction of PCa RFS in TCGA-PRAD. The effective and robust predictability of this model was validated in six independent datasets. Moreover, ENZ-sig showed a high correlation with cell cycle pathway, which may be utilized in clinic to accurately predict RFS and provided combination therapy strategies of patients with PCa.

## Data availability statement

The original contributions presented in the study are included in the article/**Supplementary Material**. Further inquiries can be directed to the corresponding authors.

## Author contributions

YF: software, formal analysis, investigation, writing—original draft, and visualization. ZT: methodology and resources. YD: visualization and resources. SC: data curation and writing—review and editing. RL: resources and data curation. JL: data curation and validation. JW: methodology and visualization. HH: formal analysis and resources. GZ: data curation. JY: investigation, resources, writing—original draft, and funding acquisition. ZH: conceptualization, methodology, project administration, writing—review and editing, and funding acquisition. WZ: conceptualization, supervision, project administration, writing—original draft, writing—review and editing, and funding acquisition. All authors contributed to the article and approved the submitted version.

## References

[1] Siegel RL Miller KD Fuchs HE Jemal A. Cancer statistics, 2022. CA: Cancer J Clin (2022) 72(1):7–33. doi: 10.3322/caac.21708 35020204

[2] Rebello RJ Oing C Knudsen KE Loeb S Johnson DC Reiter RE. Prostate cancer. Nat Rev Dis Primers (2021) 7(1):9. doi: 10.1038/s41572-020-00243-0 33542230

[3] Desai K McManus JM Sharifi N. Hormonal therapy for prostate cancer. Endocrine Rev (2021) 42(3):354–73. doi: 10.1210/endrev/bnab002 PMC8152444 33480983

[4] Nguyen PL Alibhai SM Basaria S D'Amico AV Kantoff PW Keating NL. Adverse effects of androgen deprivation therapy and strategies to mitigate them. Eur Urol (2015) 67(5):825–36. doi: 10.1016/j.eururo.2014.07.010 25097095

[5] Chen Z Lan X Thomas-Ahner JM Wu D Liu X Ye Z. Agonist and antagonist switch DNA motifs recognized by human androgen receptor in prostate cancer. EMBO J (2015) 34(4):502–16. doi: 10.15252/embj.201490306 PMC4331004 25535248

[6] Haffner MC Zwart W Roudier MP True LD Nelson WG Epstein JI. Genomic and phenotypic heterogeneity in prostate cancer. Nat Rev Urol (2021) 18(2):79–92. doi: 10.1038/s41585-020-00400-w 33328650 PMC7969494

[7] Lim ZF Ma PC. Emerging insights of tumor heterogeneity and drug resistance mechanisms in lung cancer targeted therapy. J Hematol Oncol (2019) 12(1):134. doi: 10.1186/s13045-019-0818-2 31815659 PMC6902404

[8] Taavitsainen S Engedal N Cao S Handle F Erickson A Prekovic S. Single-cell atac and rna sequencing reveal pre-existing and persistent cells associated with prostate cancer relapse. Nat Commun (2021) 12(1):5307. doi: 10.1038/s41467-021-25624-1 34489465 PMC8421417

[9] Li R Qu H Wang S Wei J Zhang L Ma R. Gdcrnatools: An R/Bioconductor package for integrative analysis of lncrna, mirna and mrna data in gdc. Bioinf (Oxford England) (2018) 34(14):2515–7. doi: 10.1093/bioinformatics/bty124 29509844

[10] Hao Y Hao S Andersen-Nissen E Mauck WM 3rd Zheng S Butler A. Integrated analysis of multimodal single-cell data. Cell (2021) 184(13):3573–87.e29. doi: 10.1016/j.cell.2021.04.048 34062119 PMC8238499

[11] Trapnell C Cacchiarelli D Grimsby J Pokharel P Li S Morse M. The dynamics and regulators of cell fate decisions are revealed by pseudotemporal ordering of single cells. Nat Biotechnol (2014) 32(4):381–6. doi: 10.1038/nbt.2859 PMC4122333 24658644

[12] Subramanian A Tamayo P Mootha VK Mukherjee S Ebert BL Gillette MA. Gene set enrichment analysis: A knowledge-based approach for interpreting genome-wide expression profiles. Proc Natl Acad Sci United States America (2005) 102(43):15545–50. doi: 10.1073/pnas.0506580102 PMC1239896 16199517

[13] Aibar S González-Blas CB Moerman T Huynh-Thu VA Imrichova H Hulselmans G. Scenic: Single-cell regulatory network inference and clustering. Nat Methods (2017) 14(11):1083–6. doi: 10.1038/nmeth.4463 PMC5937676 28991892

[14] Zhang Y Wang D Peng M Tang L Ouyang J Xiong F. Single-cell rna sequencing in cancer research. J Exp Clin Cancer Res CR (2021) 40(1):81. doi: 10.1186/s13046-021-01874-1 33648534 PMC7919320

[15] Lewis MA Sharabash N Miao ZF Lyons LN Piccirillo J Kallogjeri D. Increased Ifrd1 expression in human colon cancers predicts reduced patient survival. Digestive Dis Sci (2017) 62(12):3460–7. doi: 10.1007/s10620-017-4819-0 PMC6167971 29094309

[16] Ren X Chen X Fang K Zhang X Wei X Zhang T. Col5a2 promotes proliferation and invasion in prostate cancer and is one of seven Gleason-related genes that predict recurrence-free survival. Front Oncol (2021) 11:583083. doi: 10.3389/fonc.2021.583083 33816226 PMC8012814

[17] Kohrt SE Awadallah WN Phillips RA 3rd Case TC Jin R Nanda JS. Identification of genes required for enzalutamide resistance in castration-resistant prostate cancer cells in vitro. Mol Cancer Ther (2021) 20(2):398–409. doi: 10.1158/1535-7163.Mct-20-0244 33298586 PMC7867613

[18] Wang D Jiao Z Ji Y Zhang S. Elevated Tuba1a might indicate the clinical outcomes of patients with gastric cancer, being associated with the infiltration of macrophages in the tumor immune microenvironment. J gastrointestinal liver diseases: JGLD (2020) 29(4):509–22. doi: 10.15403/jgld-2834 33331338

[19] Tian Y Wang J Wen Q Gao A Huang A Li R. The significance of tumor microenvironment score for breast cancer patients. BioMed Res Int (2022) 2022:5673810. doi: 10.1155/2022/5673810 35528180 PMC9071896

[20] Fink EE Moparthy S Bagati A Bianchi-Smiraglia A Lipchick BC Wolff DW. Xbp1-Klf9 axis acts as a molecular rheostat to control the transition from adaptive to cytotoxic unfolded protein response. Cell Rep (2018) 25(1):212–23.e4. doi: 10.1016/j.celrep.2018.09.013 30282030 PMC6251307

[21] Larson SR Chin J Zhang X Brown LG Coleman IM Lakely B. Prostate cancer derived prostatic acid phosphatase promotes an osteoblastic response in the bone microenvironment. Clin Exp metastasis (2014) 31(2):247–56. doi: 10.1007/s10585-013-9625-2 PMC3946934 24242705

[22] Sobala ŁF Fernandes PZ Hakki Z Thompson AJ Howe JD Hill M. Structure of human endo-α-1,2-Mannosidase (Manea), an antiviral host-glycosylation target. Proc Natl Acad Sci United States America (2020) 117(47):29595–601. doi: 10.1073/pnas.2013620117 PMC7703563 33154157

[23] Barrett CS Millena AC Khan SA. Tgf-β effects on prostate cancer cell migration and invasion require fosb. Prostate (2017) 77(1):72–81. doi: 10.1002/pros.23250 27604827 PMC5286811

[24] Kwon OK Ha YS Lee JN Kim S Lee H Chun SY. Comparative proteome profiling and mutant protein identification in metastatic prostate cancer cells by quantitative mass spectrometry-based proteogenomics. Cancer Genomics Proteomics (2019) 16(4):273–86. doi: 10.21873/cgp.20132 PMC6609260 31243108

[25] Hooi CF Blancher C Qiu W Revet IM Williams LH Ciavarella ML. St7-mediated suppression of tumorigenicity of prostate cancer cells is characterized by remodeling of the extracellular matrix. Oncogene (2006) 25(28):3924–33. doi: 10.1038/sj.onc.1209418 16474848

[26] Hu D Jiang L Luo S Zhao X Hu H Zhao G. Development of an autophagy-related gene expression signature for prognosis prediction in prostate cancer patients. J Trans Med (2020) 18(1):160. doi: 10.1186/s12967-020-02323-x PMC7137440 32264916

[27] Mei W Jia X Xin S Liu X Jin L Sun X. A N(7)-Methylguanine-Related gene signature applicable for the prognosis and microenvironment of prostate cancer. J Oncol (2022) 2022:8604216. doi: 10.1155/2022/8604216 35602299 PMC9122703

[28] Feng D Xiong Q Zhang F Shi X Xu H Wei W. Identification of a novel nomogram to predict progression based on the circadian clock and insights into the tumor immune microenvironment in prostate cancer. Front Immunol (2022) 13:777724. doi: 10.3389/fimmu.2022.777724 35154101 PMC8829569

[29] Karanika S Karantanos T Li L Wang J Park S Yang G. Targeting DNA damage response in prostate cancer by inhibiting androgen receptor-CDC6-ATR-Chk1 signaling. Cell Rep (2017) 18(8):1970–81. doi: 10.1016/j.celrep.2017.01.072 PMC5349188 28228262

[30] Bridges KA Hirai H Buser CA Brooks C Liu H Buchholz TA. MK-1775, a novel Wee1 kinase inhibitor, radiosensitizes p53-defective human tumor cells. Clin Cancer Res (2011) 17(17):5638–48. doi: 10.1158/1078-0432.CCR-11-0650 PMC3167033 21799033

